# Genome-wide transcriptome analysis revealed organelle specific responses to temperature variations in algae

**DOI:** 10.1038/srep37770

**Published:** 2016-11-24

**Authors:** HyeonSeok Shin, Seong-Joo Hong, Chan Yoo, Mi-Ae Han, Hookeun Lee, Hyung-Kyoon Choi, Suhyung Cho, Choul-Gyun Lee, Byung-Kwan Cho

**Affiliations:** 1 Department of Biological Sciences, Korea advanced institute of Science and Technology, Daejon 305-701, Republic of Korea; 2 Department of Biological Engineering, Inha University, Incheon 402-751, Republic of Korea; 3 College of Pharmacy, Gachon University, Incheon 406-840, Republic of Korea; 4 College of Pharmacy, Chung-Ang University, Seoul 156-756, Republic of Korea

## Abstract

Temperature is a critical environmental factor that affects microalgal growth. However, microalgal coping mechanisms for temperature variations are unclear. Here, we determined changes in transcriptome, total carbohydrate, total fatty acid methyl ester, and fatty acid composition of *Tetraselmis* sp. KCTC12432BP, a strain with a broad temperature tolerance range, to elucidate the tolerance mechanisms in response to large temperature variations. Owing to unavailability of genome sequence information, *de novo* transcriptome assembly coupled with BLAST analysis was performed using strand specific RNA-seq data. This resulted in 26,245 protein-coding transcripts, of which 83.7% could be annotated to putative functions. We identified more than 681 genes differentially expressed, suggesting an organelle-specific response to temperature variation. Among these, the genes related to the photosynthetic electron transfer chain, which are localized in the plastid thylakoid membrane, were upregulated at low temperature. However, the transcripts related to the electron transport chain and biosynthesis of phosphatidylethanolamine localized in mitochondria were upregulated at high temperature. These results show that the low energy uptake by repressed photosynthesis under low and high temperature conditions is compensated by different mechanisms, including photosystem I and mitochondrial oxidative phosphorylation, respectively. This study illustrates that microalgae tolerate different temperature conditions through organelle specific mechanisms.

Microalgae show high growth rates and photosynthetic efficiency that greatly exceed that of current terrestrial crops as a potential source for sustainable bioenergy and biochemicals^1^,^2^. However, current levels of biomass production from microalgae in industrial scale outdoor culture systems does not match their potential due to the unstable environmental factors, such as light and temperature^3^. To overcome this, strategies to improve outdoor culture systems have been developed by improving culturing facilities that enable the reduction of photo-inhibition and heat stress by diffusing strong light with stacked reactors^1^,^4^. However, the problem of daily and seasonal temperature fluctuation remains unsolved, despite modelling studies that indicate temperature is the most critical factor for microalgal growth in outdoor culture systems^2^,^3^,^5^,^6^. The modelling and experimental data indicate that a 10 °C decrease in temperature would reduce the growth rate to half. Moreover, the growth rate of microalgae cultured in higher temperature compared to the optimal temperature decrease more rapidly and halts growth^2^,^3^. For example, *Asterionella formosa* and *Nannochlorposis oceanica* cultured in 10 °C higher temperature condition than the optimal temperature results in the reduction of their growth rate to the level of less than 10%. Considering that temperature is an uncontrollable environmental factor that strongly affects microalgae, a more fundamental understanding of the effect on microalgae is required.

Current understanding of the effect of temperature on microalgal growth is mostly focused on the effect of temperature on photosynthesis-related mechanisms. The reduced growth rate of microalgae in low temperatures is explained by reduced enzymatic activities related to the Calvin cycle and inhibition of the repair process of the D1 protein of photosystem II (PSII) which leads to photo-inhibition^5^,^7^.

Furthermore, changes in the fatty acid compositions occur in the direction of increased unsaturated fatty acid levels, which compensate for the decreased thylakoid membrane fluidity in cold temperatures^8^,^9^. Alternatively, the growth inhibition of microalgae in high temperatures is explained by a decrease in the units of the PSII complex, such as the oxygen-evolving complex of PSII and photochemical reaction centre^10^,^11^. In addition, a decrease in ribulose-1,5-bisphosphate carboxylase (RuBisCO) activity, another major enzyme involved in the carbon fixation, contributes to decreased growth rates in heat stress^12^,^13^. Thus, the temperature effect on microalgae growth rates is described as a bell-shaped curve with a gradual increase in growth rate below optimal temperature and a steep decline above optimal temperature^5^. Although these studies showed the inhibitory effect of cold and heat temperature stresses on photosynthetic mechanisms, it is unclear how microalgae adapt to temperature variations.

Therefore, we used *Tetraselmis* sp. KCTC12432BP, a strain with a broad range of temperature tolerance, to investigate the coping mechanisms of microalgae to different temperatures. Compared to the generally stated tolerance range of microalgae which is between 15 and 25 °C, *Tetraselmis* sp. exceeds both the low and high temperature tolerance range, which makes it a suitable candidate to investigate both heat and cold temperature stresses^5^,^14^. The growth, total carbohydrate content, fatty acid methyl ester (FAME) content, and fatty acid composition were compared for *Tetraselmis* sp. cultivated under different temperature conditions. Protein-coding sequences were assembled using *de novo* transcriptome assembly, which was subsequently used for differential expression analysis to explain the algal response to different temperature conditions. The integrated analysis showed different coping mechanisms of *Tetraselmis* sp. in response to temperature variation. The localization of the differentially expressed genes (DEGs) indicated an organelle level response for energy metabolism where photosynthesis is not functioning properly. Overall, this study provided a comprehensive analysis of *Tetraselmis* sp. to both cold and heat stress conditions for an enhanced understanding of its response to fluctuating temperature.

## Results

### Effect of temperature on cell growth and cellular content

**Figure 1:**
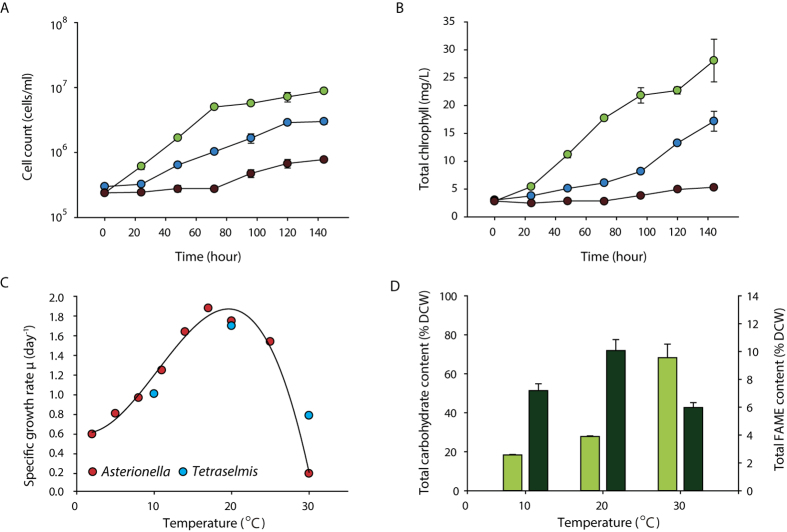
Effect of changes in temperature on *Tetraselmis* growth and cellular content. The cell count (**A**) and chlorophyll level (**B**) of *Tetraselmis* cultures in 10 °C, 20 °C and 30 °C are shown by blue, green and dark red circles, respectively. (**C**) The specific growth rate of *Tetraselmis* and *Asterionella Formosa* cultured in different temperature are shown by red and blue circles, respectively. The polynomial regression curve was drawn using the specific growth rate of *Asterionella formosa*^5^. (**D**) The total total carbohydrate content and total fatty acid methyl ester (FAME) content of the *Tetraselmis* cultures in different temperatures are shown by light and dark green bars, respectively.

To investigate the effect of different temperatures on cell growth, *Tetraselmis* sp. KCTC12432BP was cultivated in low- (10 °C), mid- (20 °C), and high- (30 °C) temperature conditions. Cells cultured in mid-temperature conditions showed higher growth rates than those in low- and high-temperature conditions, which was subsequently considered as the optimal growth condition and used as the control condition for further analyses (Fig. 1A, B). Interestingly, the cell count and amount of chlorophyll indicate that the cells cultured in the high-temperature conditions showed slower growth rates than those cultured in low-temperature conditions. Such a steep decrease of the growth rate above optimal temperatures has been previously reported in different microalgae^12^. For example, the specific growth rate of *Asterionella formosa,* a strain with a broad temperature tolerance range, showed very similar growth patterns according to the growth temperatures. The growth rate of *Tetraselmis* sp. was 0.81, 1.50, and 0.59 μ per day for low, mid, and high-temperature conditions, respectively, which concurred with the regression curve plotted from the specific growth rate of *A. formosa* (Fig. 1C)^5^. These results suggest that *Tetraselmis* sp. has a broad range of temperature tolerance as anticipated by the temperature fluctuation in isolated locations (Supplemental Fig. S1).

Microalgae cultures under stress conditions accumulate carbon in the form of storage compounds such as carbohydrate or lipid^15^. To investigate the effect of temperature stress on *Tetraselmis*, we measured the total carbohydrate and FAME contents. Compared to the cells cultured in mid-temperature, the total carbohydrate content increased and decreased by 39.1% and 9.5% for low- and high-temperature cultures, respectively (Fig. 1D). These results are similar to the stress response of other microalgae such as *Chlamydomonas reinhardtii* and *Dunaliella tertiolecta*, where nutrition deficient stress led to carbohydrate accumulation^16^,^17^. On the other hand, the FAME content of the cells cultured in low and high temperature decreased by 2.9% and 4.1% for cells cultured in low and high temperature respectively, compared to the cells cultured in mid- temperature condition (Fig. 1D). The accumulation of carbon storage differ in that both carbohydrate and FAME content decreased in low temperature while in high temperature conditions, carbohydrate content increased and FAME content decreased. These results suggests the involvement of a different stress response mechanism for different temperature.

### Effect of temperature on fatty acid composition

Microalgal fatty acid compositions change with adaptation to different temperatures^18^,^19^. To investigate the effect of different temperatures on the fatty acid composition, the fatty acids from each culture were analysed by gas chromatography. The dominant fatty acids were C16:0, C16:4, and C18:3, accounting for 73.7, 64.2, and 58.1% of the total fatty acid of *Tetraselmis* sp. cultured in low, mid, and high-temperature conditions, respectively (Table 1). These results correlate with the carbon chains of fatty acids produced by general microalgae species, which is dominant in C16 and C18^20^. Furthermore, different types of fatty acid compositions were observed under different temperature conditions. When the temperature decreased, the total polyunsaturated fatty acids (PUFA), such as C16:4 and C18:3, increased, whereas the saturated fatty acid (C16:0) decreased proportionally. The level of long chain fatty acids (C20) increased in proportion in 10 °C to 9.0% compared to 5.1% and 5.9% of 20 °C and 30 °C, respectively. These results correlate with previous studies that showed an increase in unsaturated fatty acid in response to low temperature to avoid a decrease in membrane fluidity^18^,^21^,^22^. The increase of long chain fatty acids and PUFA levels in low-temperature conditions suggests changes in the structure of membrane-forming lipids, which changed to spatially larger forms to increase membrane fluidity^21^.

### *De novo* transcriptome assembly and functional annotation

To gain insights into the underlying mechanisms triggering the phenotypic changes to the temperature variation, the transcriptome was sequenced and subsequently used to identify the functionally relevant genes. Owing to the unavailability of its genome sequence, we performed *de novo* transcriptome assembly. A total of six strand-specific sequencing libraries were generated for the three temperature conditions with biological replicates (Supplemental Table S1) and sequenced with an Illumina platform with an average output of 2.7 Gb. The reads were trimmed with the quality score of 0.05 and pooled together for *de novo* transcriptome assembly^23^. The assembly resulted in 425,485 transcripts with an average length of 356 bp and an N50 value of 574 bp (Supplemental Table S2). To confirm the amount of sequence reads used in the assembly procedure, the RNA-seq reads were mapped back to the assembled transcripts. Over 84% of the reads were mapped to the assembled transcripts for all conditions (Supplemental Table S3). To remove any false positives that may have occurred from the large input data of 16.0 Gb, the assembled transcripts were subjected to BLASTX analysis against the non-redundant (Nr) database^24^,^25^. After a stringent *E*-value cutoff of less than 10^−10^, the length coverage of 80% was used to obtain the full-length transcripts^17^. These stringent cutoff values resulted in a reduction of the total assembled bases to 31.5 Mb with 26,245 transcripts remaining (Supplemental Table S2). To test whether these stringent cutoff values resulted in a loss of data, the assembled transcripts were matched to the highly conserved 248 core eukaryotic genes (CEGs), which is used as a standard to evaluate assembly results^26^. The 26,245 transcripts that remained after cutoff matched 245 CEGs out of 248, indicating that a sufficient level of transcripts were found in the assembly results. Furthermore, the average length of the assembled transcripts showed a threefold increase compared to the whole transcripts without cutoff treatment (Supplemental Table S3). The assembled transcripts comprised 20,979 components that corresponded to the predicted genes. Among them, 17,793 were singleton genes and 3,206 genes had an average of 2.64 isoforms per gene (Table 2). Thus, further analysis was carried out using the identified 20,979 genes (26,245 transcripts).

The results of the functional annotation indicated that 48.9% of the transcripts were hypothetical or uncharacterized (Supplemental Fig. S2A)^25^. These results are possibly owing to an explosive increase in the protein sequence database. For example, the taxon distribution of the BLASTX results shows that species hit have been diversified compared to previous studies, which was dominant in *Volvox carteri, Chlorella variabilis*, and *Chlamydomonas reinhardtii* (Supplemental Fig. S2B)^17^,^27^. To address this, further analysis to identify the function of each transcript was carried out by additional BLASTX runs to the modified version of the UniProt and tremble databases, which does not contain any hypothetical protein^28^. In addition, these matches were used to annotate the transcripts with corresponding KEGG orthology (KO) ID and gene ontology consortium (GO) ID^29^,^30^. Overall, out of 20,979 genes, 16,269 and 17,564 genes were annotated with GO and KO, respectively (Supplemental Table S4).

### Changes in the energy process in response to temperature variation

**Figure 2:**
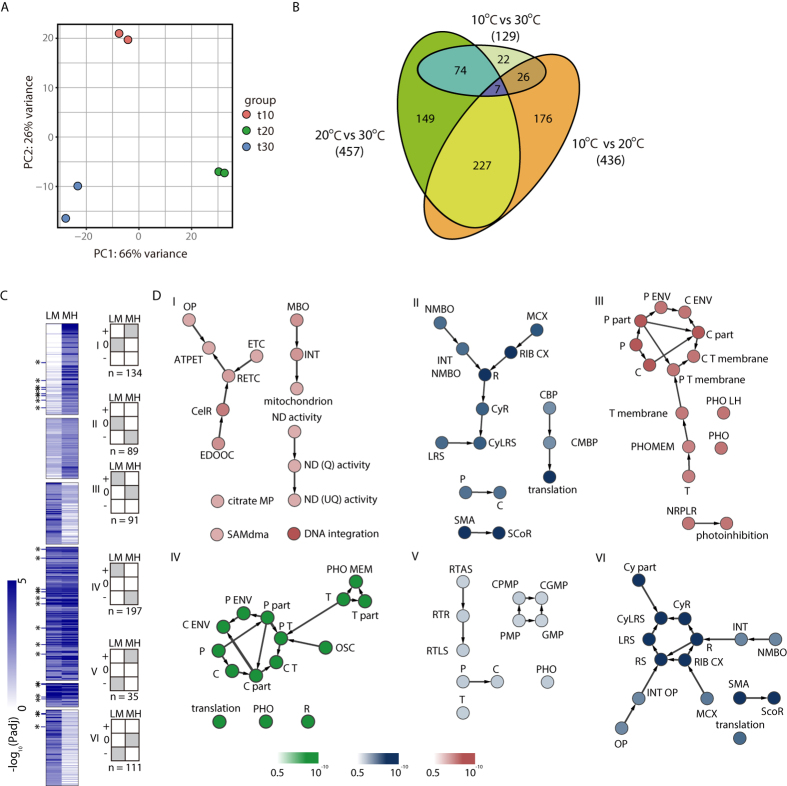
Analysis of the DEGs. (**A**) PCA plot of the 500 genes with the highest variances. (**B**) Venn diagram of the DEGs with a Padj value cutoff of 0.01 in different temperature comparisons^52^. (**C**)The heatmap showing the negative log_10_ Padj value of DEGs are shown for the six major clusters generated by the fold change pattern. The six major clusters are indicated by the grey coloured heatmap, which indicate the fold change direction for M/L and H/M, respectively. A fold-change value of <2 or Padj value >0.01 was considered as unchanged for this cluster generation. (**D**) The GO network of the top 15 enrichments of each cluster that satisfy a significant P < 0.05 of the hypergeometric test with FDR correction from BiNGO. The blue and red colours indicate enrichment of genes that are upregulated against high and low temperature, respectively. The GO terms that are upregulated in mid temperature are shown in green. ATPET, ATP synthesis coupled electron transport; C, chloroplast; CBP, cellular biosynthetic process; CelR, cellular respiration; CGMP, cellular glucan metabolic process; CMBP, cellular macromolecule biosynthetic process; CPMP, cellular polysaccharide metabolic process; CX, complex; Cy, cytosolic; ENV, envelope; ETC, electron transport chain; GMP, glucan metabolic process; INT, intracellular; LH, light harvesting; LRS, large ribosomal subunit; MBO, membrane-bound organelle; MCX, macromolecular complex; MP, metabolic process; ND, NADH dehydrogenase; NMBO, non-membrane-bound organelle; NMBO, non-membrane-bounded organelle; NRPLR, negative regulation of photosynthesis; OP, oxidative phosphorylation; OP, organelle part; OSC, organelle subcompartment; P, plastid; PHO, photosynthesis; PHOMEM, photosynthetic membrane; PMP, polysaccharide metabolic process; Q, quinone; R, ribosome; RETC, respiratory electron transport chain; RIB, ribonucleoprotein; RTAS, response to abiotic stimulus; RTLS, response to light stimulus; RTR, response to light radiation; SAMdma, S-adenosylmethionine-dependent methyltransferase activity; SCoR, structural constituent of ribosome; SMA, structural molecule activity; UQ, ubiquinone.

To determine the changes in the transcriptome to different temperature conditions, we normalized the mapped reads, which were verified by principal component analysis (PCA)^31^,^32^. All samples were grouped individually within each temperature condition, which suggested a high correlation between the biological replicates (Fig. 2A)^31^,^32^. Next, we compared the differentially expressed genes (DEGs) between temperature conditions that satisfy at least a two-fold change and an adjusted *P*-value (Padj) cutoff < 0.01. The comparisons of low to mid (LM), mid to high (MH), and low to high (LH) temperature conditions resulted in 436, 457, and 129 DEGs, respectively (Fig. 2B, Supplemental Table S5). Next, we investigated whether the DEGs were functionally enriched using a network construction of the highly enriched GO terms via BiNGO (Supplemental Table S6)^33^. Interestingly, the enrichment was differentiated at the organelle level, where the upregulated genes in low-temperature conditions were mostly localized in the plastid and the upregulated genes in high-temperature conditions were localized in the mitochondria (Supplemental Fig. S3). For the comparison of MH conditions, the downregulated genes in high-temperature conditions were mainly composed of photosynthetic, cellular biosynthetic, and metabolic processes that enable cell proliferation. Considering the previously known inhibitory effect of heat stress on photosynthetic machinery, the decline in growth rate at high temperature results from the limited rate of photosynthesis^10^,^11^. However, the upregulated genes in high-temperature conditions were enriched in oxidative phosphorylation, the respiratory electron transport chain, and NADH dehydrogenase, which generate energy in the form of NADH and ATP in the mitochondria. In particular, genes localized between the inner and outer membranes of the mitochondria, such as apocytochrome b (TR187067_c0_g1, TR77369_c0_g1), cytochrome c oxidase subunit 1 and 3 (TR273020_c0_g3, TR15219_c0_g1, TR196800_C2_G1), and core subunits of the NADH dehydrogenase (2, 4, 5, and 7) (TR185307_c0_g1, TR223398_c0_g2, TR263675_c0_g2, TR204700_c0_g8, TR220555_c0_g2), were activated, indicating that energy is highly utilized through the mitochondrial electron transport chain. This was further demonstrated in the comparison of LH conditions (Supplemental Fig. S3). The mitochondrial genes involved in energy processing were downregulated in high-temperature conditions. In addition, the functionally enriched GO terms indicated that oxidation of acetyl-CoA via the TCA cycle and cellular respiration was activated compared to that in low-temperature conditions. This data was consistent with the enrichments of the oxidative phosphorylation in the comparison of MH conditions, which generated NADH from the TCA cycle for ATP synthesis.

In the comparison of LM conditions, both up- and downregulated genes in low-temperature conditions showed localization in the plastid. The downregulation of photosynthesis-related genes was expected, but cold stress also induced the downregulation of photosynthesis. However, upregulation of plastid and chlorophyll related genes in low-temperature conditions were not anticipated (Supplemental Table S6). To verify these results, we further investigated the DEGs related to photosynthesis. The upregulated genes in low-temperature conditions comprised the photosynthetic electron transfer chain (PETC), such as the light-harvesting complex II chlorophyll a/b binding protein 1 (LHCA1) (TR275472_c0_g1), LHCA3 (TR204700_c0_g8), cytochrome b6-f complex subunits (*petC*) (TR35563_c0_g4), and ATP synthase (plastidic) (TR1667_c0_g1). The downregulated genes in low-temperature conditions comprised a broad range of photosynthesis-related genes. In particular, genes constituting PSII were enriched compared to the upregulated genes (Supplemental Table S5). These results suggest that the major machinery for the energy-harvesting complex (PSII) was downregulated, and photosystem I (PSI) and PETC were activated to reconstitute the loss in energy uptake under low-temperature conditions.

Clustering analysis is advantageous to obtain a global expression pattern of the genes because all three temperatures are considered, whereas DEG analysis considers only a pairwise comparison of two temperature conditions. Using the fold change values from the comparison of LM and MH conditions, we generated six clusters that showed distinct patterns in all three temperature conditions (Fig. 2C and Supplemental Table S7). Cluster I consisted of genes that were upregulated only in high-temperature conditions, which showed similar patterns to the upregulated genes in the high-temperature conditions in the comparison of MH conditions, as anticipated (Supplemental Fig. S4). The genes were enriched in the mitochondrion electron transport chain (METC), including nitrogen dehydrogenase with ubiquinone (UQ) activity, indicating that energy processing was uniquely shifted toward the mitochondria in high-temperature conditions (Fig. 2D). This was more evident by the different enrichment pattern in cluster V, which showed no enrichment in relation to the mitochondrion energy uptake process. Since cluster V is composed of upregulated genes under both high and low-temperature conditions and the shift in energy uptake to mitochondrion would have also occurred in low temperature, the enrichment pattern would be similar to that of cluster I. Instead, all functional enrichments in cluster V showed a very weak FDR value over 0.03 which was slightly larger than the cutoff of 0.05, indicating that the cells responded to low and high temperature stress with different stress responsive mechanisms (Supplemental Table S8).

Cluster VI showed enrichment in the cytosolic parts of the ribosomal protein subunits (Fig. 2D). The molecular mechanism of differential uses of cytosolic ribosome in response to low temperature was unclear; however, we predicted that a translation mechanism may respond to different folding of RNA. Clusters II, III, and IV showed functional enrichments of the upregulated genes under optimal temperature conditions, and their functional enrichments were localized to the plastid and chlorophyll. In particular, cluster IV contained 197 genes out of the 227 genes that were differentially expressed in the comparison of both LM and MH conditions, accounting for 53.7–51.2% of the total DEGs of LM and MH, respectively (Supplemental Fig. S3). This data clearly indicated that photosynthesis (especially the PSII complex) was downregulated in response to both cold and heat stress (Table 3). Furthermore, whereas cluster III showed a very similar enrichment pattern to cluster IV, which showed that the top 15 functional enrichments were related to photosynthesis, cluster II showed weaker enrichment to photosynthesis (Fig. 2D). This data suggests that in low-temperature conditions, some of the photosynthetic machinery remained active, which correlated with the upregulated genes in low-temperature conditions. Finally, to validate the expression level obtained from RNA-seq analysis, qRT-PCR was performed on the genes that showed different response to temperature variation. Although the fold change level by qRT-PCR for the comparison of LM showed a smaller magnitude, the overall results of RNA-seq and qRT-PCR results were well correlated (Supplemental Fig. S4).

### Effect of temperature variation on lipid structure

**Figure 3:**
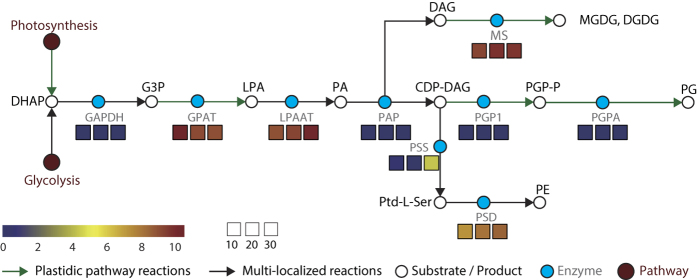
The reconstructed lipid biosynthesis pathway of *Tetraselmis* sp. KCTC12432BP. The green arrows indicate reactions that occur only in the plastid and the black arrow indicate reactions that may occur in different cell parts. The enzymes are represented by a light blue circle with grey labels, and substrate/product is represented by white circles with black labels. The colour index indicates the expression pattern of the enzymes in low mid and high temperatures. Abbreviations: DAG, diacylglycerol; DHAP, dihydroxyacetone phosphate; GAPDH, glyceraldehyde-3-phosphate dehydrogenase; G3P, glycerol 3-phosphate; GPAT, glycerol-3-phosphate acyltransferase; LPA, lysophosphatidic acid; LPAAT, lysophosphatidic acid acyltransferase; MS, monogalactosyldiacylglycerol synthase; PA, phosphatidic acid; PAP, phosphatidate phosphatase; PE, phosphatidylethanolamine; PG, phosphatidylglycerol; PGP-P, phosphatidylglycerol-phosphate; PGP1, phosphatidylglycerolphosphate synthase; PGPA, phosphatidylglycerolphosphatase A; PSS, phosphtidylserine synthase; PSD, phosphatidylserine decarboxylase; Ptd-l-Ser, phosphatidylserine.

To verify the organelle level changes under different temperature conditions, we next investigated if genes responsible for lipid structure are also differentially expressed. Since the lipid structure plays an important role in cellular metabolism and many of the differentially expressed genes were localized specifically in the thylakoid and mitochondrial membranes, we reconstructed the lipid pathway from the transcriptome assembly and analysed their expression patterns (Fig. 3). Glycerol-3-phosphate acyltransferase, which encodes the enzyme that converts glycerol 3-phosphate to lysophosphatidic acid (LPA), increased in the low-temperature conditions (TR233525_c1_g1). Furthermore, GO annotation for TR233525_c1_g1 showed that this protein was localized in the plastid, which indicated the increased LPA level in the plastid. As LPA synthesis is a rate-limiting reaction step of lipid biosynthesis in the plastid, this data suggests increased levels of galactoglycerolipids, such as MGDG and DGDG, and phosphoglycerolipids, such as PG, from the plastidic pathway^34^. However, enzymes responsible for synthesis of PE were upregulated in high-temperature conditions (Fig. 3). Since PE is the main lipid structure that composes the inner membrane of mitochondria along with phosphatidylcholine, this data suggests further proof for activation of mitochondrial energy uptake processes being activated in high-temperature conditions^35^,^36^.

## Discussion

**Figure 4:**
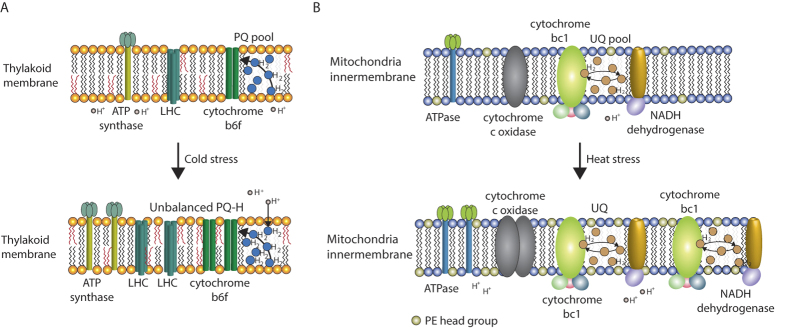
A schematic model of the response of *Tetraselmis* sp. KCTC12432BP to different temperatures. (**A**) Changes in the thylakoid membrane caused by cold stress and (**B**) changes in the mitochondrial membrane caused by heat stress. The upregulated proteins and lipid structures are presented by increased appearances.

Responses of *Tetraselmis* sp. KCTC12432BP to low temperature can be described as salvaging photosynthesis by adjusting the cellular contexts favourable for photosynthesis. First, the total FAME analysis showed an increase in unsaturated fatty acids, which have been previously explained as a response to increase thylakoid membrane fluidity that would otherwise be decreased by low temperature^8^,^9^,^37^,^38^,^39^,^40^. Furthermore, the transcriptome data showed increased lipid biosynthesis in the plastid pathway that constitutes the major lipid structure in the thylakoid membrane. Changes in the lipid structure of the thylakoid membrane affect the stabilization of the D1 protein, which is important because the repair process of D1 protein is inhibited in low temperatures resulting in reduced photosynthesis efficiency^7^,^37^,^38^,^39^,^40^. Second, the transcriptome analysis showed increased levels of several genes of the PETC (Fig. 2C). The specific genes upregulated in low temperature were LHCA1, LHCA3, cytochrome b6f, and ATP synthase, which are sequentially after PSII in the PETC. In normal conditions, PQ obtains hydrogen ions (H^+^) from the stroma and the active PSII complex generates electrons from the water, which is transferred to PQ to form plastoquinole^10^,^11^,^12^,^13^. The plastoquinole transfers H^+^ to the lumen that generates an H^+^ gradient for ATP synthesis by ATP synthase and the electron would be transferred through the PETC to generate NADPH. However, in low temperatures, the PQ pool is in a reduced state of plastoquinole owing to the decreased rate of metabolism that causes high excitation pressure (HEP) (Fig. 4A)^41^. Thus, several genes of the PETC are upregulated in the low temperature condition to compensate for the decreased electron flow by HEP. Furthermore, upregulation of LHCA proteins that belong to PSI also suggests increased cyclic electron flow from PSI for an additional energy source^42^.

According to the changes in the transcriptome, energy metabolism of *Tetraselmis* sp. KCTC12432BP in high temperatures primarily occurs in mitochondria, where the major shifts are localized in between the inner and outer membranes. This is evident by the upregulation of the ETC in mitochondrial membranes and the upregulated synthesis of PE, which is one of the major components in lipid structures that forms the inner mitochondrial membrane (Fig. 4B)^43^. This suggests that oxidative phosphorylation is activated to generate energy in the form of ATP in high-temperature conditions; however, oxidative phosphorylation requires a breakdown of a carbon source^44^. Considering that cells in high temperatures showed high levels of total carbohydrate accumulation, it is unlikely that carbohydrates would be used as the carbon source. However, the FAME content decreased in the high-temperature conditions. As beta-oxidation is the process of catabolizing fatty acids to generate energy and acetyl-CoA, evidence of beta-oxidation can explain both the low FAME content and generation of a carbon source enabling oxidative phosphorylation. Therefore, we investigated whether the main enzymes that carry out beta-oxidation were upregulated in the high-temperature condition compared to the low-temperature condtition^44^,^45^. Interestingly, we found three enzymes, acyl-CoA dehydrogenase (TR277977_c0_g3), enoyl-CoA hydratase (TR213479_c1_g1), and 3-hydroxyacyl-CoA dehydrogenase (TR107284_c0_g1) that are responsible for the first three reaction of beta-oxidation. This data suggests the possibility of a fatty acid breakdown to fuel oxidative phosphorylation in high temperatures. Although slow, *Tetraselmis* sp. KCTC12432BP in high-temperature conditions was able to sustain the growth rate at the level of approximately 40% of the growth rate of the cultures in optimal temperatures. Thus, photosynthesis was severely damaged from heat stress but was not completely shut down, as the cells were cultivated autotrophically without any carbon source. We hypothesized that the small amount of NADPH generated by reduced photosynthesis would be used to store energy in the form of starch, explaining the carbohydrate accumulation.

The difference in photosynthetic activities of *Tetraselmis* in low, mid and high temperature correlates with the transcriptome analysis results. We measured the level of dissolved oxygen as the quantification level of dissolved oxygen can count for the level of oxygen produced and consumed by photosynthesis and oxidative phosphorylation, respectively. Compared to the mid-temperature, the cultures in low temperature condition showed 19.3% decrease in the level of dissolved oxygen, while the cultures in high temperature condition showed 75.2% decrease in the level of dissolved oxygen (Supplemental Fig. S5). This data support the transcriptome data that the dramatically decrease of dissolved oxygen level account for the effect of both reduced photosynthesis and increased oxidative phosphorylation. Overall, the transcriptome, FAME, and carbohydrate analysis showed two mechanisms that *Tetraselmis* sp. KCTC12432BP used to survive, even under cold and heat stress conditions. *Tetraselmis* sp. KCTC12432BP survived in the low temperature condition by reducing the damaged photosynthesis from cold stress via increased unsaturated fatty acid biosynthesis. In high-temperature conditions, the even more damaged photosynthetic mechanisms are not salvaged; instead, the cells generate energy by oxidative phosphorylation using fatty acid degradation as a carbon source.

## Methods

### Cell culture and growth measurements

*Tetraselmis* sp. KCTC12432BP (Chlorodendraceae) was locally isolated from costal seawater of the Yellow Sea in Incheon, Korea. Duplicate cultures were grown in 0.5 L bubble column photobioreactors (BC-PBRs) containing 0.4 L of sterile three folded f/2-Si medium (Guillard and Ryther, 1962) at three temperatures 10, 20, and 30 °C. BC-PBRs were illuminated continuously with 55 W daylight fluorescent lights at 100 ± 5 μmolE/m^2^/s, which was measured using a Data Logger (LI-1400, LI-COR, Lincoln, NE, USA) with a quantum sensor (LI-190SA, LO-COR). Cultures were aerated with 2% CO_2_ balanced air at a flow rate of 0.1 vvm. Cell counts and size distributions were collected every 24 h using a Coulter Counter (Multisizer 4, Beckman Coulter Inc., Brea, CA, USA). Fresh cell weight (FCW) and dry cell weight (DCW) was calculated by the cell concentration and cell size distribution. Experimental cultures were harvested in mid exponential phases. Nitrate concentration was measured spectrophotometrically and nitrate was added in culture broth to avoid nutrient limitation. Chlorophyll content was determined spectrophotometrically by 90% methanol extraction method^46^. Briefly, the cells were collected by centrifugation and extracted with 90% methanol in the dark until the biomass appears colorless. After extraction, the cell debris was removed by centrifugation. The chlorophyll content was calculated by the following equation: Total chlorophyll content = (4.0 × A665) + (25.5 × A650).

### Fatty acid composition analysis

To analyse the fatty acid contents of *Tetraselmis* sp. KCTC12432BP, cells were collected by centrifugation at 1900 g for 5 min and washed twice with distilled water. The fatty acids from the freeze-dried cells were extracted in a mixture of acetyl chloride and methanol (5:100, *v*/*v*) with methyl nonadecanoate as the internal standard. The extracted fatty acids were analysed by gas chromatography (YL6500GC, Young Lin Inc., Anyang, Korea), with the capillary column (HP-INNOWAX, 30 m and 0.53 mm internal diameter), flame ionization detection (FID), and helium as the carrier gas. The fatty acids composition was calculated quantitatively by the internal standard method and methyl nonadecanoate and total FAME content were determined by the sum of each fatty acid content. The total carbohydrate analysis were performed with the previously described method^17^. Briefly, 0.25 mL of cell culture and 5% phenol solution were mixed in the glass vial covered with aluminium foil. The 1.25 mL of sulfuric acid was added to the glass vial and vigorously stirred for 30 sec and kept at 80 °C for 30 min. Sample absorbance was determined at 492 nm using a spectrophotometer (UV-1800, Shimadzu, Kyoto, Japan). The sample concentrations were then quantified using a calibration curve obtained with maltose under identical conditions.

### RNA extraction and RNA-seq library construction

Total RNA was extracted with some modification from a previous method^17^. Briefly, the cells were washed with the extraction buffer of 200 mM Tris-HCl pH 7.5 (Bioneer, Daejeon, Korea republic of), 25 mM EDTA (Bioneer), 250 mM NaCl (Bioneer), and 0.5% SDS (Sigma-Aldrich, St. Louis, MO, USA), which was instantly frozen using liquid nitrogen and ground into a find powder by using a pestle and mortar. The pulverized cells were immediately transferred to TRIzol reagent (Invitrogen, CA, USA) and the total RNA was purified using ethanol precipitation. The RNA-seq library was constructed using the TruSeq mRNA Sample Prep Kit (Illumina, CA, USA) according to the manufacturer’s instructions. Briefly, poly-A tailed mRNA was purified with provided oligo dT magnetic beads and fragmented for 2 min to obtain RNA fragments longer than 150 bp. cDNA was synthesized from the fragmented RNA and A-tailed to both ends of the cDNA. The A-tailed cDNA was ligated to adaptors and the properly ligated products were then enriched by polymerase chain reaction (PCR). The resulting libraries were sequenced by the HiSeq 2500 instrument using the 100 bp single-end recipe (Illumina).

### *De novo* transcriptome assembly and differential expression analysis

The *de novo* transcriptome assembly was performed with Trinity version v2.0.6 for strand-specific RNA reads with default options^23^. The assembled transcripts were subjected to BLASTX to the Nr database and further analysis was performed with fully assembled transcripts that showed an E-value and length coverage of 10^−10^ and 80%, respectively. To obtain additional information on the transcripts, the UniProt database was modified by extracting only the KO ID assigned sequences, and was subsequently used for additional functional annotation via the conversion of BLAST results to KEGG ID and GO term for further annotations into the KEGG pathway and GO network mapping^25^,^29^,^30^,^47^. The differential analysis pipeline followed the suggested pipeline for Trinity assembled transcripts^48^. Briefly, the read count was calculated using RSEM and the count data was normalized using the DESeq2 pipeline^31^,^32^. The GO enrichment analysis was conducted using the BiNGO Cytoscape application^33^. The custom annotation file was parsed with in-house script from three annotations acquired by the UniProt database. The enrichment was analysed using a hypergeometric test with an FDR corrected P-value of 0.05 as a cutoff.

### Quantitative real-time PCR (qRT-PCR) analysis

To validate the results from the RNA-seq analysis qRT-PCR was performed on some of the genes that are differentially regulated in different temperature conditions. As the housekeeping gene of *Tetraselmis* is not known, we selected *fabl* (TR222023_c0_g1_i1) as the housekeeping gene by searching the RNA-seq expression data for stable expression under different temperature conditions. The qRT-PCR was performed by constructing first strand cDNA followed by quantification using real time PCR using SYBR green. Briefly, the first stand cDNA was constructed using SuperScript III First-Strand Synthesis System (Thermo scientific, Waltham, MA) with random hexamers according to their instructions for each conditions and its biological replicates. Any residual RNA were removed by RNaseH treatment. The specific primers for each gene were designed according to the suggestion by SYBR Green Real-Time PCR Master Mix (BIO-RAD, Hercules, CA) for appropriate melting temperature around 60 °C and the product length between 90 bp to 120 bp (Supplementary Table S9).

### Measurement of the photosynthetic activity

The photosynthetic activity (PA) was obtained by measuring the change in dissolved oxygen (DO) concentration in different temperature conditions, and calculated by Eq (1) where, C_b_ is the biomass concentration and O_2_ is the DO concentration^49^.


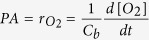


Probe for DO (InPro6850i, Mettler Toledo, Urdorf, Switzerland) was connected to a data acquisition system (M300 ISM, Mettler Toledo, Urdorf, Switzerland) and DO concentration was measured as previously described^50^. Briefly, the oxygen probe was one-point calibrated with a 100% air saturation to measure the oxygen production rate. The samples were prepared by using a 0.1 g DCW/L of cells which were already acclimated to the different temperatures. The cultures were taken to the 500 mL photobioreactor to avoid side effects such as mutual shading, nutrient deficiency and photo-oxidative stress. The light was turned on after they were dark-acclimated and the oxygen production was measured for 20 min. The dissolved oxygen concentration was obtained in 1 min intervals and expressed as percent (%) air saturation. The conversion to milligram per liter was calculated based on Henry’s law, thus, adjusting the maximum solubility of oxygen in water for the temperature effect: 100% air saturation equals a dissolved oxygen concentration of 11.3 mg/L, 9.1 mg/L and 7.5 mg/L at 10 °C, 20 °C and 30 °C^51^.

## Additional Information

**How to cite this article**: Shin, H. S. *et al*. Genome-wide transcriptome analysis revealed organelle specific responses to temperature variations in algae. *Sci. Rep.* **6**, 37770; doi: 10.1038/srep37770 (2016).

**Publisher’s note:** Springer Nature remains neutral with regard to jurisdictional claims in published maps and institutional affiliations.

## Supplementary Material

Supplementary Information

Supplementary Table 4

Supplementary Table 5

Supplementary Table 6

Supplementary Table 7

Supplementary Table 8

## Figures and Tables

**Table 1:** Fatty acid composition of *Tetraselmis* sp. KCTC12432BP in different temperature conditions.

	Temperature		
	10 °C	20 °C	30 °C
C16:0	20.23 ± 2.41	21.76 ± 0.77	32.49 ± 0.02
C16:2	3.67 ± 0.05	2.59 ± 0.15	1.82 ± 0.06
C16:4	20.67 ± 4.34	18.56 ± 0.56	9.80 ± 0.42
C18:0	0.63 ± 0.44	0.32 ± 0.01	1.37 ± 0.21
C18:1	5.06 ± 0.01	9.21 ± 0.07	13.89 ± 0.43
C18:2	1.17 ± 0.02	7.97 ± 0.62	15.71 ± 0.87
C18:3	32.78 ± 3.61	23.87 ± 4.98	15.77 ± 0.04
C18:4	6.79 ± 2.93	10.67 ± 2.96	3.24 ± 0.52
C20:1	3.90 ± 0.19	1.08 ± 0.09	1.95 ± 0.21
C20:4	0.90 ± 0.55	1.17 ± 0.27	nd
C20:5	4.20 ± 0.09	2.81 ± 0.29	3.95 ± 0.45
ΣSFA	20.86	22.07	33.86
ΣMUFA	8.97	10.29	15.84
ΣPUFA	70.17	67.64	50.30

Abbreviation: SFA, saturated fatty acid; MUFA, mono-unsaturated fatty acid; PUFA, poly-unsaturated fatty acid; nd, non-detected.

**Table 2:** Statistics of assembled transcriptome of *Tetraselmis* sp. KCTC12432BP.

Statistics of the assembly	
Total number of transcripts	26,245
Number of genes	20,980
Number of singleton genes	17,793
Number of genes with predicted isoforms	3,206
Number of transcripts in isoforms	8,472
Average number of isoforms for genes with isoform	31.5 (Mbp)

**Table 3:** The downregulated genes in low temperature that are involved in photosystem II in the comparison of LM.

Assembly	FC(LM)*	FC(MH)**	20 °C
TR94846_c0_g1	−6.99	7.09	psbW; photosystem II PsbW protein
TR94843_c1_g1	−4.03	5.89	LHCB5; light-harvesting complex II chlorophyll a/b binding protein 5
TR256172_c3_g1	−4.12	3.31	LHCB5; light-harvesting complex II chlorophyll a/b binding protein 5
TR255926_c2_g1	−3.93	3.80	LHCB4; light-harvesting complex II chlorophyll a/b binding protein 4
TR224453_c2_g1	−4.30	6.10	PRK, prkB; phosphoribulokinase [EC:2.7.1.19]
TR222288_c1_g1	−3.98	4.65	psbR; photosystem II 10 kDa protein
TR221127_c1_g1	−5.23	5.02	psbP; photosystem II oxygen-evolving enhancer protein 2
TR205377_c0_g1	−3.87	5.54	:GAPA; glyceraldehyde-3-phosphate dehydrogenase (NADP + ) (phosphorylating) [EC:1.2.1.13]
TR149089_c0_g1	−5.67	7.21	psbM; photosystem II PsbM protein

^*^FC(LM) is the log_2_ fold change value of the comparison of LM, where negative value indicate upregulation of low temperature.

^**^FC(LM) is the log_2_ fold change value of the comparison of MH, where positive value indicate upregulation of mid temperature.
