# Supplementary material for: Genome-wide transcriptome analysis revealed organelle specific responses to temperature variations in algae
Source: Sci Rep. 2016 Nov 24;6:37770. doi: 10.1038/srep37770 (PMC5121895; doi:10.1038/srep37770)
Supplement: Supplementary Information [file srep37770-s1.pdf]

Genome-wide transcriptome analysis revealed organelle specific response to temperature variation in algae

HyeonSeok Shin[1,¶], Seong-Joo Hong[2,¶], Chan Yoo[1], Mi-Ae Han[2], Hookeun Lee[3], Hyung-Kyoon Choi[4], Choul-Gyun Lee[2], and Byung-Kwan Cho[1,*]

[1]Department of Biological Sciences, Korea advanced institute of Science and Technology, Daejon 305-701, Republic of Korea

[2]Department of Biological Engineering, Inha University, Incheon 402-751, Republic of Korea

[3]College of Pharmacy, Gachon University, Incheon 406-840, Republic of Korea

[4]College of Pharmacy, Chung-Ang University, Seoul 156-756, Republic of Korea

*Correspondence and requests for materials should be addressed to B.-K.C. (bcho@kaist.ac.kr)

[¶]These authors equally contributed to this work.

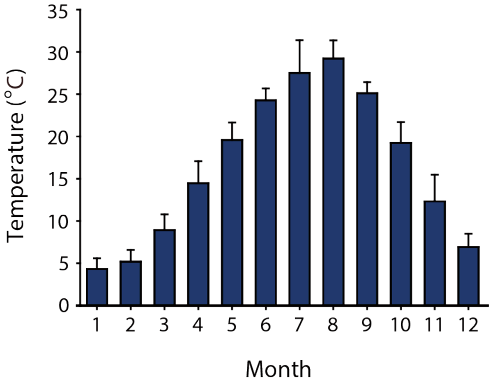

Supplemental Figure S1. The temperature fluctuation of the west sea of Korea. Measurements from Augustus of 2013 to Augustus of 2014 were obtained from Korea Hydrographic and Oceanographic Agency.

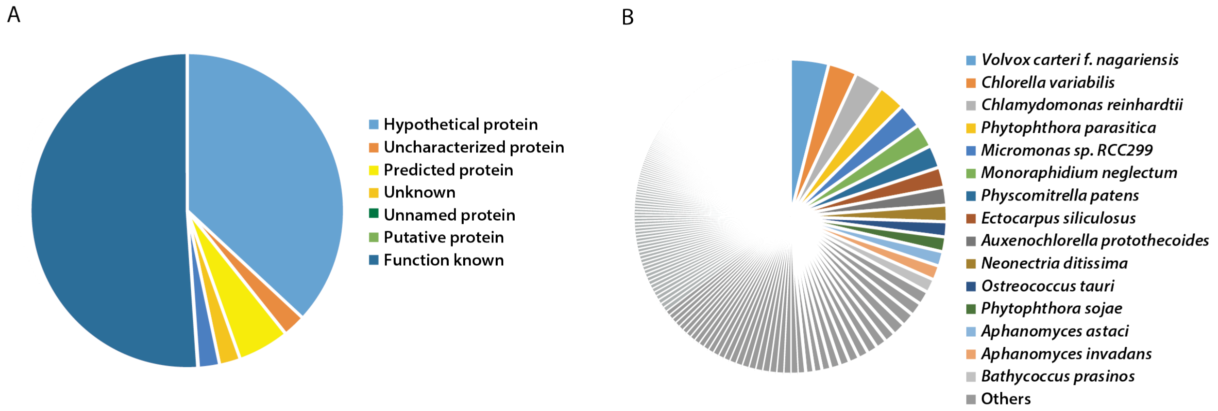

Supplemental Figure S2. Annotation statistics from Nr database. (A) Pie chart showing the distribution of the functional description of the subject protein. (B) Pie chart showing the taxon hit distribution of the subject protein. Only the top 15 BLAST hits are colored and labeled. The taxon distribution that is not within top 15 are colored with dark grey and unlabeled.

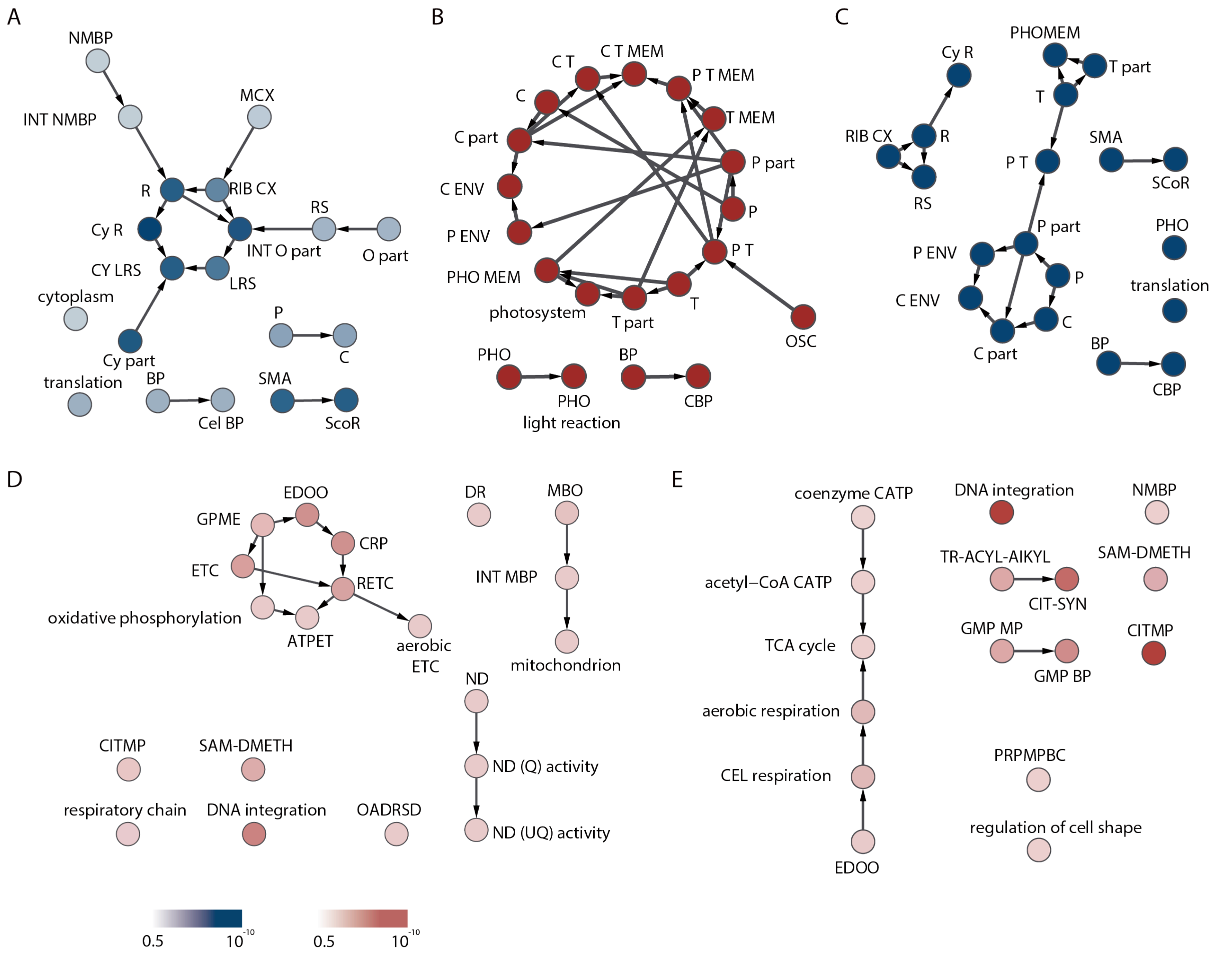

Supplemental Figure S3. DEG analysis via GO network enrichment. The top 20 GO enrichment terms that satisfy a significant P-value < 0.05 of hypergeometric test with FDR correction from BiNGO are shown with circular YFiles layout. For M vs L, (A) the upregulated genes in L and (B) the upregulated genes in M are shown. For H vs M, (C) the upregulated genes in M and (D) the upregulated genes in H are shown. For H vs L, the upregulated genes in H are shown in (E), however no enrichments were found for upregulated genes in L. The blue color indicate the upregulated conditions have a lower temperature to the compared condition and the red color indicate the upregulated conditions have a higher temperature to the compared condition. ATPET, ATP synthesis coupled electron transport; BP, biosynthetic process; C, chloroplast; CATP, catabolic process; Cel, cellular; CITMP, citrate metabolic process; CIT-SYN, citrate (SI)-synthase activity; CRP, cellular respiration; Cy, cytosolic; DR,defense response; EDOO, energy derivation by oxdiation of organic compounds; ENV, envelope; ETC, electron transport chain; GPME, generation of precursor metabolites and energy; INT, intracellular; LRS, large ribosomal subunit; MEM, membrane; ND, NADH dehydrogenase; NMBP, nucleoside monophosphate biosynthetic process; O, organelle; OADRSD, oxidoreductase activity, acting on dphenols and related substances as donors; OSC, organelle subcompartmnet; P, plastid; PRPMPBC, positie regulation of protein maturation by peptide bond cleavage; Q, quinone; R, ribosome; RS, ribosomal subunit; SAM-DMETH, S-adenosylmethionine-dependent methyltransferase activty; SCoR,

structural constituent of ribosome; SMA, structural molecule activity; TCA, tricarboxylic acid; TR-ACYL-AlKYL, transferase activity, transferring acyl groups, acyl groups converted into alkyl on transfer; UQ, ubiquinone;

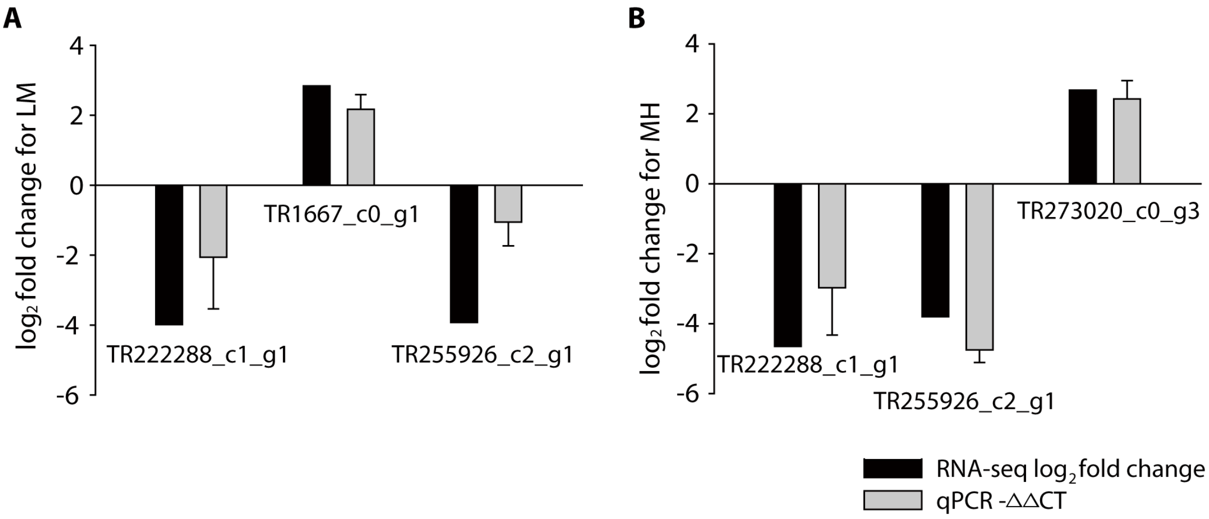

Supplemental Figure S4. The relative fold change level of RNA-Seq and qRT-PCR. The $\log_2$ fold change is shown in black bar and the C_T value of qRT-PCR is shown in grey bar for (a) low temperature vs mid temperature (LM) and (b) mid temperature vs high temperature.

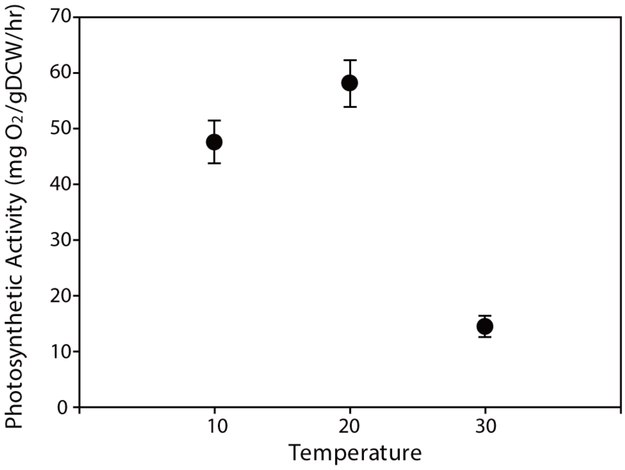

Supplemental Figure S5. Measurement of the dissolved oxygen level in varying temperature conditions.

Supplemental Table S1. Transcriptome sequencing results *Tetraselmis* sp. KCTC12432BP

	10°C	20°C	30°C
Replicate 1	2.51 Gb	3.11 Gb	2.90 Gb
Replicate 2	2.12 Gb	2.74 Gb	2.96 Gb
Trimmed replicate 1	2.32 Gb	2.94 Gb	2.59 Gb
Trimmed replicate 2	1.96 Gb	2.59 Gb	2.69 Gb

Supplemental Table S2. Assembly statistics of *de novo* transcriptome assembly of *Tetraselmis* sp. KCTC12432BP

Trinity results of Tetraselmis	Before cutoff	After cutoff
Total number of assembled Trinity transcripts	425,485	26,245
Total Number of Trinity predicted genes	359,844	20,979
Average length of the transcripts	356 bp	1,199 bp
Total assembled bases	217 Mbp	31.5 Mbp
CEG	248	245

Supplemental Table S3. Percentage of the mapped reads to Trinity assembly

Temperature	10°C	20°C	30°C
**Replicate1**	91.62%	84.34%	90%
**Replicate 2**	90.38%	86.1%	84.92%

Supplemental Table S4, S5, S6, S7 and S8 are provided in separate excel sheets at http://www.nature.com/srep.

Supplemental Table S9. Primers used in qRT-PCR

Gene	Transcript	Direction	Primer sequence (5 to 3)
fabI	TR222023_c0_g1_i1	Forward	GCCTTCTTGCCTGTCAAATCG
fabI	TR222023_c0_g1_i1	Reverse	GTCCTGGGTGCGCAGAAA
psbR	TR222288_c1_g1_i1	Forward	ACAGGGAGTTAGGGTTTGGTC
psbR	TR222288_c1_g1_i1	Reverse	GTCTCCTTGTTGTCGGCCTC
atpF	TR1667_c0_g1_i1	Forward	TGTTTCTTTAGGTGGTGATGCC
atpF	TR1667_c0_g1_i1	Reverse	CTAACGCTAAACGAGCACGAG
*Lhcb*4	TR255926_c2_g1_i1	Forward	GCATGCTATCTAGTGCTTTGCT
*Lhcb*4	TR255926_c2_g1_i1	Reverse	GCGTCCTTGGCTCCCTG
*Cox*1	TR273020_c0_g3_i1	Forward	TGGGGCCTTTTCTGGAGTTTTA
*Cox*1	TR273020_c0_g3_i1	Reverse	CGTTATAAAGTTGGTGGTTTCCTCC
